# Test–retest reliability of performance variables during treadmill rollerski skating

**DOI:** 10.1007/s00421-025-05746-w

**Published:** 2025-03-27

**Authors:** Thomas Losnegard, Paul André Solberg, Magne Lund-Hansen, Martin Skaugen, Joar Hansen, Knut Skovereng, Øyvind Sandbakk

**Affiliations:** 1 Department of Physical Performance, The Norwegian School of Sport Sciences, Ullevål Stadion, Post Box 4014, 0806 Oslo, Norway; 2 Norwegian Olympic Federation, Oslo, Norway; 3 Section of Health and Exercise Physiology, Inland Norway University of Applied Science, Lillehammer, Norway; 4 Department of Neuromedicine and Movement Science, Faculty of Medicine and Health Sciences, Center for Elite Sports Research, Norwegian University of Science and Technology, Trondheim, Norway; 5 School of Sport Science, UiT The Artic University of Norway, Tromsø, Norway

**Keywords:** Oxygen uptake, Performance, Training, Nordic skiing, Cross-country skiing

## Abstract

**Purpose:**

We examined the test–retest reliability of rollerski testing across a familiarization trial followed by three separate test trials (T1–T3) conducted within a 14-day period.

**Methods:**

Ten competitive cross-country skiers performed three sub-maximal tests (5%, speed range 10–16 km h^−1^) and a maximal speed test until failure (MTF; ~ 5–8 min, 7%, > 10 km h^−1^) on a rollerski treadmill using the Gear 3 ski skating sub-technique. Reliability was assessed as within-subject typical error, expressed as a coefficient of variation (CV%, [confidence limits]) intraclass correlation (ICC, [confidence limits]), and changes in mean (%).

**Results:**

The speed at MTF demonstrated a mean CV (T1–T3) of 1.5% [1.1, 2.6] and an ICC of 0.96 [0.87, 0.99], but a systematic familiarization bias from T1 to T2 (1.2% [0.1, 2.3]) and T2 to T3 (2.2% [0.1, 4.3]). Peak oxygen uptake exhibited a mean CV of 2.2% [1.6, 3.8] and an ICC of 0.93 [0.78, 0.98], with no systematic changes from T1 to T2 (− 0.2% [− 2.0, 1.6]) and T2 to T3 (1.8% [− 1.1, 4.7]). VO_2_ at submaximal load showed a mean CV of 2.1% [1.5, 3.3] and an ICC of 0.94 [0.84,0.99], with no systematic changes from T1 to T2 (− 0.7% [− 2.4, 1.1]) and T2 to T3 (− 0.1% [− 2.4, 2.3]).

**Conclusion:**

The relatively low CV and high ICC for most measures suggest a high degree of test–retest reliability. However, the systematic mean changes in MTF indicate that familiarization trials are essential to provide valuable information about individual changes. Overall, these reliability measures can be used as a framework by practitioners to discern true changes when testing on a rollerski treadmill.

**Supplementary Information:**

The online version contains supplementary material available at 10.1007/s00421-025-05746-w.

## Introduction

Physiological variables that determine performance are often evaluated by both researchers and coaches to provide diagnostic information about training-induced changes. In most endurance sports, laboratory tests are conducted in a “sport-specific” manner, aimed at identifying precise training-induced adaptations. However, reliable results are crucial for the valid interpretation of data from such physiological tests. According to Hopkins (2000) three crucial measures of reliability should be quantified: within-subject variation, retest correlation, and changes in the mean. When a subject undergoes multiple tests, random variation between trials can occur, observed as the standard deviation of individual values. This within-subject variation, also known as typical error, is expressed as the coefficient of variation (CV) of measurement.

Cross-country skiing, biathlon, and Nordic combined are Olympic sports that utilize the freestyle skiing technique, also known as ski skating. Similar to other endurance sports, higher aerobic metabolic energy turnover (i.e., peak oxygen uptake (VO_2peak_) and its fractional utilization) and/or a reduced cost of locomotion (i.e., enhanced work economy/efficiency) are the primary drivers of performance responses and are, therefore, commonly measured test values. In these sports, large rollerski treadmills are used for “sport-specific” testing, which allows skiers to replicate their skiing technique and simulate on-snow skiing accurately from a biomechanical perspective (Myklebust et al. 2014, 2022).

Physiological testing on rollerskis has been extensively utilized for decades (Hoffman et al. 1994; Holmberg et al. 2005; Sandbakk et al. 2010; Losnegard et al. 2013) yet the learning effect and typical error over multiple tests have not been thoroughly examined. Such information is crucial when determining training-induced changes or evaluating experimental interventions. However, to date, only two studies have investigated the test–retest reliability of performance and physiological measurements during treadmill skiing. Losnegard et al. (2013) reported the CV in VO_2peak_ (2.3%), O_2_-cost (1.2%), and 1000-m time-trial performance (2.7%) while rollerski skating on a treadmill. Bucher et al. (2023) conducted a test–retest reliability study of a comprehensive test battery, including a VO_2max_ test using the diagonal stride technique and a 24-min time-trial test while double poling on a treadmill. The CV was 1.4% for VO_2peak_ and 1.0% for the 24-min time trial. However, since these previous studies only included two trials, less is known about possible learning effects over multiple trials within a short testing period.

Cross-country skiing is performed at varying speeds and inclines due to significant variations in terrain. From a testing perspective, this means that the most relevant inclines and speeds must be covered. Nevertheless, there is general agreement that moderate uphill terrain is particularly relevant for testing, considering the importance of uphill performance, the avoidance of excessively high speeds indoors where air drag is absent, and the induction of competition-relevant speeds during both submaximal and maximal testing (Sandbakk et al. 2010; Losnegard et al. 2013; McGawley and Holmberg 2014; Andersson et al. 2016). On such inclines, the Gear 3 skating sub-technique (i.e., synchronized pole plants for every ski push-off) is the most used sub-technique during races and testing (Andersson et al. 2010; Sandbakk et al. 2011; Sollie et al. 2021).

The aim of the present study was to examine the test–retest reliability of performance-determining variables from submaximal and maximal tests using the skating technique during treadmill rollerskiing. We chose a protocol with a constant incline and increasing speed, in which within-subject variation, test–retest correlation, and changes in the mean were investigated.

## Methods

### Participants

Four female and six male competitive cross-country skiers were recruited (age range 20–30 years). The participants were categorized as Tier 3 according to McKay et al. (2022). All subjects were familiar with testing and training on a rollerski treadmill, but not specifically with the protocols used in the present study. This was a retrospective study based on pre-existing data collected during 2020–2022 at the Norwegian Olympic training centers (Oslo, Trondheim, and Lillehammer, Norway) and informed written consent was obtained from all subjects.

### Experimental design

All participants performed a total of four tests within 14 days, where Test 0 (T0) was the familiarization to the test and T1 to T3 was used for establishing the reliability of the test-protocol. During all tests, participants performed three submaximal bouts each of 5 min duration and one maximal trial, conducted as a time to failure test. Oxygen uptake (VO_2_), heart rate (HR), blood lactate concentration (La^−^) and rate of perceived exertion (RPE) were measured during all trials. Two participants were tested in the early preparation phase (June), while the remaining 6 participants were tested in the late preparation phase (September–October). The 14-day testing period was planned based on each participant’s periodic plan to assure appropriate training load. The participants were told not to have strenuous activity the days prior to each test, otherwise, the training was not controlled.

### Protocol and measurements

After a standardized ten-minute warm-up consisting of low-intensity skiing, participants completed three sub-maximal bouts at 5% incline and individualized speed (initial start speed of 10–12 km∙h ^−1^), each lasting five minutes with 2 min break. The intensity of each load was individually increased by adjusting speed in increments of 1 or 2 km∙h^−1^ which was identical for all four test occasions. Submaximal assessments included the measurement of steady-state VO_2_, HR, and La^−^. Submaximal O_2_-cost (VO_2sub_) was defined as the average oxygen uptake (in milliliters per kilogram per minute) between 2.5 and 4.5 min at each bout and the average of all bouts were used for data analyses for each test. Heart rate was measured in the same 2-min period and the average of the 3 bouts was used for data analyses. Reported RPE and a blood sample for the evaluation of La^−^ was taken 30 s after each bout. Eight minutes after the submaximal trial, participants performed a maximal test at 7% incline. Treadmill performance (indicated by maximal test to failure; MTF) was assessed with an incremental treadmill test. The initial speed was individually set (10–13 km∙h^−1^) was increased by 1 km∙h^−1^ every minute until failure. The performance (MTF) was determined as the calculated maximal speed (Speed_max_), by using the speed of the last completed 60-s stage plus the number seconds on the following stage. Further, the numbers of remaining seconds were divided by 60 s. Hence, if participants had a MTF of 6 min and 30 s and the last completed 60 s was 17 km h^−1^ their Speed_max_ was calculated as 17.5 km h^−1^. Gas exchange and HR were measured continuously. The average of the three highest consecutive 10-s measurements of VO_2_ was designated as VO_2peak_ (in milliliters per kilogram per minute). RPE was reported 1 min after the test.

### Apparatus

All tests were performed on a rollerski treadmill (Rodby, Södertalje, Sweden or Forcelink B.V., the Netherlands). $\dot{V}O_{2}$ was measured using an automatic ergospirometry system with a mixing chamber setup (Oxycon Pro; Jaeger Instruments, Höchberg, Germany or Vyntus CPX Vyaire Medical, Chicago, Il, USA). La^−^ was measured using a Biosen C-Line GP + lactate analyzer (EKF Diagnostic, Cardiff, United Kingdom). All participants wore the same pair of roller skis from test to test (Swenor Long skate, Sarpsborg, Norway or IDT, Lena, Norway, with a coefficient of rolling resistance; C_rr_ = 0.014–0.018) and NNN ski binding (Rottefella, Lier, Norway). The participants used regulation length poles of type Swix Triac 3.0 (Swix Sport, Lillehammer, Norway). The participants wore their personal ski boots and HR monitor. Body mass was measured using an electronic body mass scale (Seca model nr: 877; Seca GmbH & Co., Hamburg, Germany). During all test, each participants used the same equipment (treadmill, ergospirometry, etc.).

### Statistical analysis

Data are shown as means and standard deviations. To analyze changes in the mean from Test 0 (familiarization) to Test 3, a one-way analysis of variance (ANOVA) with repeated measures was conducted. If a main effect was identified, Bonferroni post hoc pairwise comparisons were applied. A *P*-value of less than 0.05 was considered statistically significant. To assess reliability across testing time points, a spreadsheet for calculating the coefficient of variation (CV), intraclass correlation coefficient (ICC; 3,1), and change in mean presented in relative terms (percentage change) was used (Hopkins 2015). To assess the standardized size of the CV we used the following scale: 0.1–0.3: small; 0.3–0.6: moderate; 0.6–1.0: large (Smith and Hopkins 2011). Statistical calculations were performed using Microsoft Office Excel 2013 (Microsoft, Redmond, WA) and SigmaPlot software (version 14.0; Systat Software Inc, San Jose, CA).

## Results

### Change in the mean

**Fig. 1:**
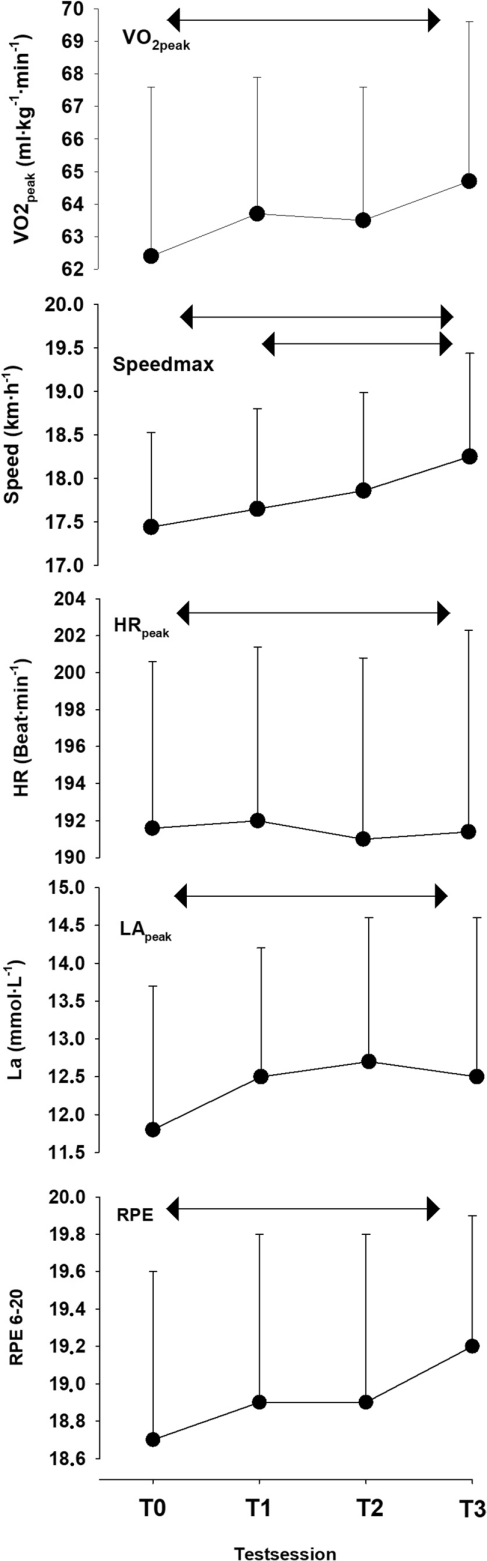
Absolute change score with standard deviation for the maximal test (**a**) and submaximal test (**b**). T0 is the familiarization test, T1–T3 are tests 1–3. Arrows show the area of statistical differences from a one-way ANOVA with repeated measures test (*P* < 0.05)

Overall changes during submaximal and maximal tests from T0 to T3 are illustrated in Fig. 1, while specific % time-point changes, excluding T0, from T1–T2 and T2–T3 are shown in Table 1. The one-way ANOVA revealed that Speed_max_ significantly increased from T0 to T3 (3.4% [1.8, 5.1], *P* < 0.01), and post hoc analyses indicated a significant difference between T1 and T3 (*P* < 0.01), with no significant differences observed between the other tests. VO_2peak_ showed a significant increase from T0 to T3 (4.1% [1.2, 7.1], *P* = 0.04), but no significant differences between the other tests. HR_sub_ decreased from T0 to T3 (− 2.7% [− 1.6, − 3.8], *P* < 0.01), with post hoc analyses also showing significant differences between T0 and T2, as well as T1 and T3 (both *P* < 0.05). Both La^−^ and RPE decreased from T0 to T3 (both *P* < 0.01), with no significant differences found between the other tests. VO_2Sub_ did not exhibit any significant changes across the tests.

**Table 1:** Mean change (%) for Speed_max_, VO_2peak_, peak heart rate (HR_peak_) at maximal test to failure and submaximal O_2_-cost (VO_2sub_) and heart rate (HR_sub_) during submaximal loads from T1 to sT3

	Speed_max_	VO_2peak_	HR_peak_	VO_2sub_	HR_sub_
	% change (95% CI)	% change (95% CI)	% change (95% CI)	% change (95% CI)	% change (95% CI)
*T1*–*T2*	1.2 (0.1–2.3)	– 0.2 (– 2.0, 1.6)	– 0.5 (– 1.6, 0.5)	– 0.7 (– 2.4, 1.1)	– 1.8 (– 3.4, – 0.2)
*T2*–*T3*	2.2 (0.1, 4.3)	1.8 (– 1.1, 4.7)	0.2 (– 0.7, 1.1)	– 0.1 (– 2.4, 2.3)	– 0.9 (– 2.9, 1.2)

CI = Confidence Interval

### Typical error and intraclass correlation

Typical error expressed as coefficient of variation and intraclass correlation are shown in Tables 2 and 3. The typical errors were relatively stable and small to moderate for Speed_max_, VO_2peak_, VO_2Sub_, with typically small typical error T1–T2. For HR_peak_ the typical errors were small T1–T2 and T2–T3, while they were moderate for HR_Sub_.

**Table 2:** Coefficient of variation (%) and intraclass correlation (ICC) for MTF and peak oxygen uptake (VO_2peak_) and peak heart rate (HR_peak_) during the maximal test conducted at 7% from T1 to T3

	Speed_max_		VO_2peak_		HR_peak_	
	CV	ICC	CV	ICC	CV	ICC
*T1*–*T2*	1.0 (0.7, 1.9)	0.98 (0.93, 0.99)	1.7 (1.1, 3.2)	0.96 (0.82, 0.99)	1.0 (0.6, 1.8)	0.98 (0.89, 0.99)
*T2*–*T3*	1.9 (1.3, 3.7)	0.94 (0.75, 0.99)	2.6 (1.8, 5.1)	0.90 (0.63, 0.98)	0.8 (0.6, 1.6)	0.98 (0.93, 1.0)
*Mean T1*–*T3*	1.5 (1.1, 2.6)	0.96 (0.87, 0.99)	2.2 (1.6, 3.8)	0.93 (0.78, 0.98)	0.9 (0.7, 1.5)	0.98 (0.93, 1.00)

Data are mean and (95CL; lower, upper)

**Table 3:** Coefficient of variation (%) and intraclass correlation (ICC) for physiological variables for submaximal O_2_-cost (VO_2sub_) and heart rate (HR_sub_) during submaximal loads conducted at 5% from T1 to T3

	VO_2sub_		HR_sub_	
	CV	ICC	CV	ICC
*T1*–*T2*	1.7 (1.2, 3.2)	0.96 (0.85, 0.99)	1.6 (1.1, 2.9)	0.93 (0.75, 0.98)
*T2*–*T3*	2.3 (1.6, 4.3)	0.92 (0.76, 0.98)	2.1 (1.4, 3.9)	0.85 (0.50, 0.96)
*Mean T1*–*T3*	2.1 (1.5, 3.3)	0.94 (0.76, 0.98)	2.0 (1.4, 3.3)	0.90 (0.69, 0.97)

Data are mean and (95CL; lower, upper)

## Discussion

The current study evaluated the test–retest reliability of a commonly used rollerski protocol, characterized by a constant incline and escalating speed. This procedure included one familiarization session (T0) and three trials (T1–T3) within a 14-day period for skiers who were already familiar with the rollerski treadmill, though not specifically with this protocol.

The principal findings include:

1. Speed_max_ revealed a mean CV from T1–T3 of 1.5% [1.1, 2.6] and an ICC of 0.96 [0.87, 0.99], with a systematic familiarization bias from T0–T3 (3.4% [1.8, 5.1]) and T1–T3 (both *P* < 0.05) with a change in T1 to T2 of 1.2% [0.1, 2.3] and from T2 to T3 of 2.2% [0.1, 4.3].
2. VO_2peak_ showed a mean CV of 2.2% [1.6, 3.8] and an ICC of 0.93 [0.78, 0.98], with a with a systematic familiarization bias from T0–T3 (4.1% [1.2, 7.1], *P* < 0.05), while no clear detectable systematic familiarization bias from T1 to T2 (− 0.2% [− 2.0, 1.6]) or T2 to T3 (1.8% [− 1.1, 4.7]).
3. Submaximal O_2_-cost (VO_2sub)_ showed a mean CV of 2.1% [1.5, 3.3] and an ICC of 0.94 [0.84, 0.99], with no clear systematic familiarization bias from T0–T3 with T1 to T2 of − 0.7% [− 2.4, 1.1] or T2 to T3 of − 0.1% [− 2.4, 2.3].

Graded exercise tests have faced criticism for their reliability and ecological validity (McGawley 2017; Currell and Jeukendrup 2008). In the present study, it is evident that a significant familiarization effect occurs, with Speed_max_ continuing to increase through the fourth test (Fig. 1). The test, which lasted approximately 6–7 min, demonstrated a mean increase of about 3% in Speed_max_ from T1 to T3, suggesting a systematic familiarization bias. The ~ 3% increase in Speed_max_ from T1 to T3 coincided with no changes in VO_2peak_, HR_peak_, La^−^_peak_, and RPE_peak_ across the three main tests. Consequently, the skiers appeared to reach the same level of "exhaustion" at the end of the MTF tests as well as similar peak physiological variables. Despite the systematic familiarization bias in Speed_max_, the relatively low CV (1.5% from T1 to T3) and high ICC (0.96 from T1 to T3) similar to what was found in Losnegard et al. (2013), suggest a high degree of test–retest reliability.

McGawley (2017) found that the VO_2max_ was higher during a graded running test than during a time-to-failure test, suggesting that VO_2max_ can vary depending on the test type. In the preliminary data collection (see Supplemental Data) we investigated differences between increased incline and increased speed and found no absolute differences in VO_2peak_, HR_peak_ or RPE_peak_. Moreover, Losnegard et al. (2012a) found no difference in ski skating VO_2max_ between a graded test and a time-to-failure test similar to the one used in the present study. In the present study, the T1–T3 CV of VO_2peak_ was 2.2%, and HR_peak_ was 0.9%, comparable to earlier studies in running (McGawley 2017) and in the skating technique (Losnegard et al. 2013), but slightly higher than the diagonal style (1.5%) assessed by Bucher et al. (2023) using Douglas Bags. Taken together, based on the current knowledge on rollerski testing, different types of protocols for addressing VO_2peak_ imply a typical CV of VO_2peak_ on rollerskiing of 1.5–2.5% while the tested types of protocol do not seem to have a major influence on the CV in VO_2peak_.

Importantly, cross-country skiing is an intermittent sport involving a variety of speeds and techniques. When selecting a specific testing protocol for skiers, the purpose of the test and the targeted qualities must be considered. Previous studies have generally opted for increased speed (Sandbakk et al. 2010; McGawley and Holmberg 2014; Losnegard et al. 2017b), increased incline (Losnegard et al. 2012b; Pellegrini et al. 2011) or a combination of both (Kvamme et al. 2005; Gløersen et al. 2020; Andersson et al. 2016). In a preliminary study (see Supplementary Material) we examined the changes in the mean and CV of two different protocols over two tests, either by increasing the incline (with constant speed) or by increasing the speed (with constant incline). We concluded that both protocols showed similar CV and changes in the mean for submaximal variables, while the CV was larger for a maximal speed test to failure and for a VO_2peak_ test with gradually increasing speed. Combined with the clear familiarization effect from the main project, this suggests that technique is a major determinant of performance in such tests. Therefore, when planning testing, it is essential to consider the main purpose of the test (e.g., physiological assessment versus technique and high-speed qualities).

VO_2sub_ and HR_sub_ showed a T1–T3 CV of approximately 2%, comparable to previous findings in rollerski skating (Losnegard et al. 2013). This suggests that submaximal testing at aerobic steady-state speeds is suitable for detecting relatively minor changes in work economy/efficiency and heart rate. This is crucial not only for monitoring training-induced changes (Losnegard et al. 2013) but also for detecting technical alterations (Losnegard et al. 2017a) or changes in equipment (Losnegard et al. 2017b). However, despite the learning effect appearing minor in VO_2sub_ (e.g., % change), a reduced HR_sub_, and La^−^_sub_ were found between T0 and T3. Therefore, we emphasize the importance of familiarization, not only with skiing on the treadmill but also within the specific test setting (e.g., using a mouthpiece and safety harness), to ensure the most reliable data.

### Methodical considerations

In the current study, we selected highly trained skiers (Tier 3), similar to the study by Bucher et al. (2023). The participants’ level can potentially influence reliability, as better skiers typically have less variation in performance than slower counterparts (Spencer et al. 2014). However, the CV between tests was similar to what was found in elite skiers (Losnegard et al. 2013). Additionally, we included both female and male participants, which likely have influenced the relatively high ICC due to heterogeneity in the sample.

Our design involved four tests within a 14-day period to avoid significant training-induced changes. This design seems appropriate for detecting reliability over a short time-period (such as familiarization before a research project), but it might not necessarily provide the “correct” reliability of testing over longer periods. Of note is the maximal test, where subjects likely “remembered” how many speed changes they performed in the previous trial and aimed to improve their performance. Over a longer period, this learning effect might diminish.

Reliability depends on several factors, including biological and psychological factors, equipment, testing staff, and environmental conditions. It is important to acknowledge that the testing was conducted in three different labs with different equipment, testing staff, and facilities (e.g., treadmill and ergospirometry). Moreover, the participants were tested in different time-periods during the preparations phase, and the training prior and under the 14 days were not controlled. Although our standardized procedures were identical, these factors could have influenced the results and should be considered when interpreting the findings. However, the main purpose was to identify within-subject reliability over several tests, and we believe that the present setup is suitable for this objective.

## Conclusion

The relatively low CV and high ICC for most measures suggest a high degree of test–retest reliability. However, the current study found systematic changes in the mean for the MTF test, indicating that familiarization trials are essential to provide valuable information about individual changes over a short time-period. Overall, the reliability measures presented here can be used as a framework by practitioners to address true changes from measurement variability when testing on a rollerski treadmill.

## Supplementary Information

Below is the link to the electronic supplementary material.

Supplementary file1 (DOCX 25 KB)

## Data Availability

The datasets generated and analysed during the current study are available from the corresponding author on reasonable request.
