# Correction: Test–retest reliability of performance variables during treadmill rollerski skating

**DOI:** 10.1007/s00421-025-05809-y

**Published:** 2025-06-04

**Authors:** Thomas Losnegard, Paul André Solberg, Magne Lund-Hansen, Martin Skaugen, Joar Hansen, Knut Skovereng, Øyvind Sandbakk

**Affiliations:** 1 Department of Physical Performance, The Norwegian School of Sport Sciences, Ullevål Stadion, Post Box 4014, 0806 Oslo, Norway; 2 Norwegian Olympic Federation, Oslo, Norway; 3 Section of Health and Exercise Physiology, Inland Norway University of Applied Science, Lillehammer, Norway; 4 Department of Neuromedicine and Movement Science, Faculty of Medicine and Health Sciences, Center for Elite Sports Research, Norwegian University of Science and Technology, Trondheim, Norway; 5 School of Sport Science, UiT The Artic University of Norway, Tromsø, Norway

**Correction: European Journal of Applied Physiology** 10.1007/s00421-025-05746-w

In the original version of this article, figure B was missing in Fig. 1.

Figure 1 which previously appeared as

**Figure:**
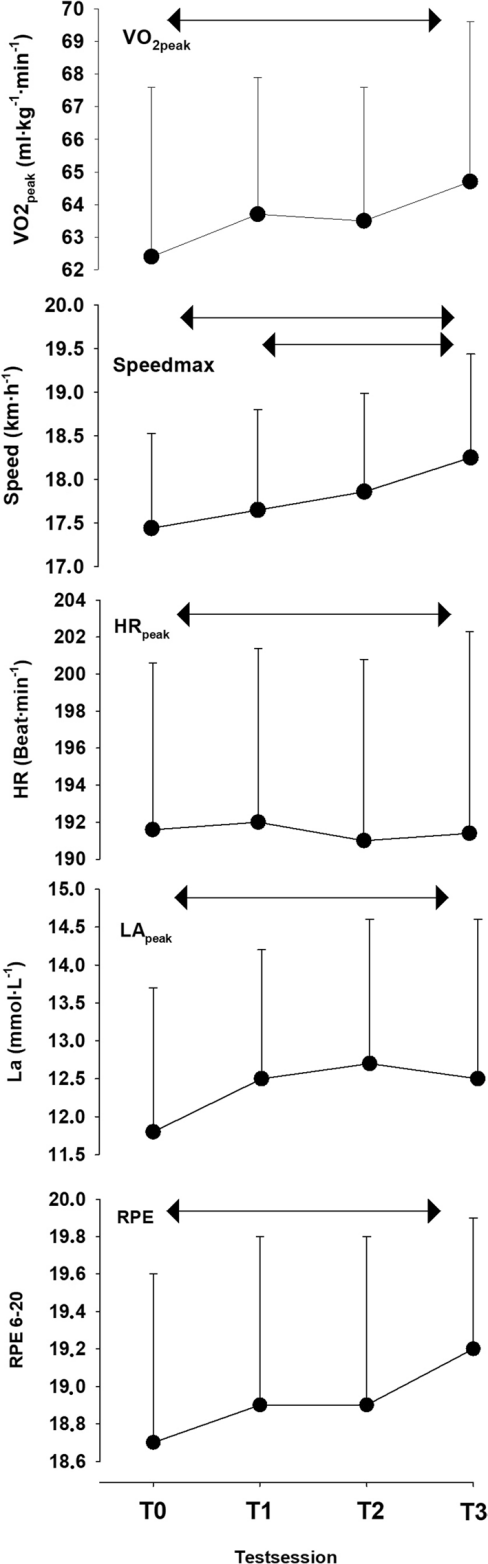
**Fig. 1** Absolute change score with standard deviation for the maximal test (**a**) and submaximal test (**b**). T0 is the familiarization test, T1–T3 are tests 1–3. Arrows show the area of statistical differences from a one-way ANOVA with repeated measures test (*P* < 0.05)

but should have appeared as shown below.

**Fig. 1:**
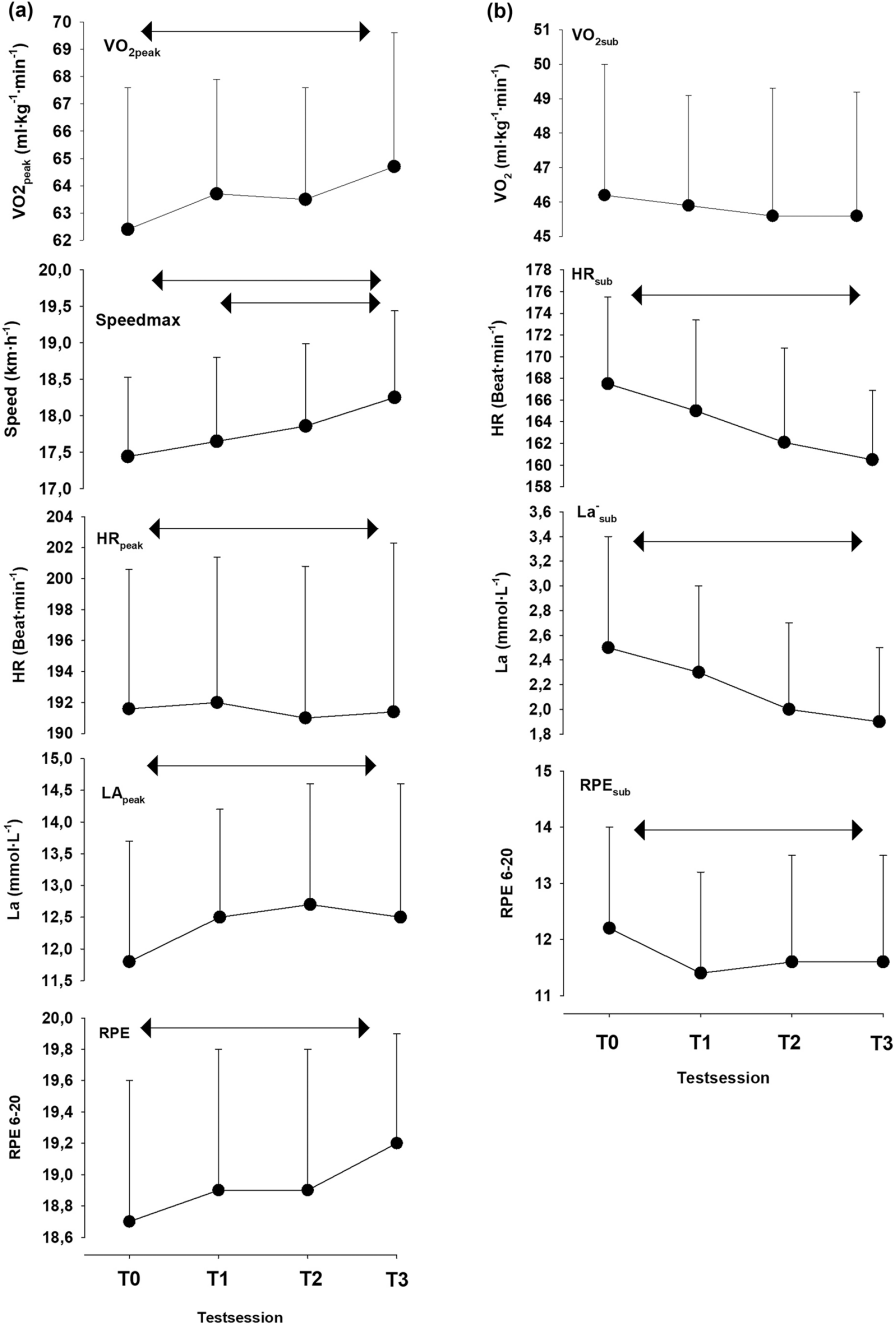
Absolute change score with standard deviation for the maximal test (**a**) and submaximal test (**b**). T0 is the familiarization test, T1–T3 are tests 1–3. Arrows show the area of statistical differences from a one-way ANOVA with repeated measures test (*P* < 0.05)

