# Supplementary material for: Test–retest reliability of performance variables during treadmill rollerski skating
Source: Eur J Appl Physiol. 2025 Mar 27;125(7):2015–21. doi: 10.1007/s00421-025-05746-w (PMC12227345; doi:10.1007/s00421-025-05746-w)
Supplement: Supplementary file 1 — Supplementary file1 (DOCX 25 KB) [file 421_2025_5746_MOESM1_ESM.docx]

**Supplemental data**

1. **Purpose:** This preliminary study examined the test-retest reliability of a rollerski test-protocol performed as either an increase in incline or an increase in speed.
2. **Methods:** Two female and six male competitive cross-country skiers were recruited. All subjects were familiar with testing and training on a rollerski treadmill, but not specifically with the protocols used in the present study. All participants performed a total of three tests within 14 days, where Test 0 (T0) was the familiarization to the test and T1 to T2 was used for establishing the test-retest reliability. All testing was conducted with the G3 (V2) ski skating technique. On T1 and T2, with a counter balanced fashion, participants underwent a protocol with *“Increasing speed”* or a protocol with *“Increasing incline”*. During both tests, participants performed 3-5 submaximal trials each of 5 min duration and one maximal trial, conducted as a time to failure test. For maximal test, *“Increasing incline”* and “*Increasing speed”* were performed on both T1 and T2 with a 30 min break (15 min passive and 10 min active recovery) between tests in a counter balanced fashion. Oxygen consumption, heart rate, blood lactate concentration and rate of perceived exertion were measured during all trials (see main project for details). For *“Increasing incline”*, speed was set to 3 m·s^-1^ and the incline was increased by 0.5˚ every submaximal stage with an individual starting incline. At the maximal test, starting incline was individualized and the incline was increased 0.5˚ every minute to failure. For *“Increasing speed”*, incline was set on 3˚ and speed increased by 0.5 m·s^-1^ for every submaximal stage and every minute during the maximal test. We estimated the maximal speed or incline (Speed_max_, Incline_max_)” where we used the average speed/incline of the last minute of the test plus the remaining seconds of the last speed/incline.
3. **Results:** Table 1-3 shows the test-retest change in the mean, CV and ICC for the two conditions; “*Increasing speed”* and *“Increasing incline”.* Peak physiological values for VO_2_, HR and RPE are shown in Table 4.
4. **Conclusion:** The performance measure during “*Increasing speed”* show slightly higher CV, % mean change and lower ICC than *“Increasing incline”*. Physiological factors did not show any major differences at sub- or maximal protocol between the protocols.

Table 1: Test-retest mean change (%) for Speed_max_ and Incline_max_, VO_2peak_, Heart rate peak (HR_peak_) at maximal test to failure and VO_2_ sub and HR sub for test during “Increasing speed” and “Increasing incline”. VO_2_ are absolute values (ml·min^-1^). N=8

|  | **Speed_max_ / Incline_max_** | **VO_2peak_** | **HR_peak_** | **VO_2_ Sub** | **HR Sub** |
| --- | --- | --- | --- | --- | --- |
|  | **% change**  **(95% CI)** | **% change**  **(95% CI)** | **% change**  **(95% CI)** | **% change**  **(95% CI)** | **% change**  **(95% CI)** |
| *Inc. incline* | -0.8 (-2.3, 0.7) | -0.5 (-3.0, 2.0) | -0.3 (-1.3, 0.7) | -0.4 (-2.0, 1.4) | -1.2 (-4.8, 2.5) |
| *Inc. speed* | 2.1 (-0.7, 4.9) | 0.2 (-2.8, 3.4) | 0.1 (-1.1, 1.4) | -1.5 (-3.4, 0.4) | -2.0 (-5.9, 2.2) |

Table 2: Test-retest coefficient of variation (CV;%) an intraclass correlation for Speed_max_ and Incline_max_ and corresponding physiological variables VO_2_ and heart rate (HR) during the maximal tests for “Increasing speed” and “Increasing incline”. Data are mean and (95CL; lower, upper). VO_2_ are absolute values (ml·min^-1^). N=8

|  | **Speed_max_ / Incline_max_** | | **VO_2peak_** | | **HR_peak_** | |
| --- | --- | --- | --- | --- | --- | --- |
|  | **CV** | **ICC** | **CV** | **ICC** | **CV** | **ICC** |
| *Inc.incline* | 1.2 (0.7, 2.6) | 0.99 (0.95, 1.00) | 2.0 (1.3, 4.4) | 0.98 (0.86, 1.00) | 0.8 (0.5, 1.7) | 0.99 (0.95, 1.0) |
| *Inc.speed* | 2.3 (1.5, 4.8) | 0.89 (0.57, 0.98) | 2.6 (1.7, 5.4) | 0.95 (0.70, 0.99) | 1.0 (0.7, 2.1) | 0.98 (0.89, 1.0) |

Table 3: Test-retest coefficient of variation (CV;%) an intraclass correlation (ICC) for VO2 and heart rate (HR) during the submaximal test conducted for “Increasing speed” and “Increasing incline”. VO_2_ are absolute values (ml·min^-1^). Data are mean and (95CL; lower, upper). N=8

|  | **VO_2_ sub** | | **HR sub** | |
| --- | --- | --- | --- | --- |
|  | **CV** | **ICC** | **CV** | **ICC** |
| *Inc. incline* | 1.5 (1.0, 3.0) | 0.99 (0.97, 1.00) | 3.2 (2.1, 6.6) | 0.77 (0.21, 0.95) |
| *Inc. speed* | 1.7 (1.1, 3.5) | 0.99 (0.96, 1.00) | 3.5 (2.3, 7.3) | 0.88 (0.51, 0.97) |

Table 4: Peak physiological values ( VO_2peak_, HR_peak_)and perceived exertion (RPE) during test 1 and 2 for “Increasing speed” and “Increasing incline”. VO_2_ are absolute values (ml·min^-1^). N=8

|  | **VO_2peak_ (L·min^-1^)** | **HR_peak_ (beat ·min^-1^)** | **RPE_peak_ (0-10)** |
| --- | --- | --- | --- |
|  | **Mean ± SD** | **Mean ± SD** | **Mean ± SD** |
| *Inc. incline 1* | 5.20 ± 0.50 | 189 ± 9 | 9 ± 1 |
| *Inc. incline 2* | 5.17 ± 0.43 | 189 ± 10 | 9 ± 1 |
| *Inc. speed 1* | 5.22 ± 0.50 | 188 ± 10 | 9 ± 1 |
| *Inc. speed 2* | 5.25 ± 0.38 | 189 ± 11 | 9 ± 1 |
